# Effects of sensorimotor training volume on recovery of sensorimotor function in patients following lower limb arthroplasty

**DOI:** 10.1186/s12891-015-0644-9

**Published:** 2015-08-19

**Authors:** Torsten Pohl, Torsten Brauner, Scott Wearing, Knut Stamer, Thomas Horstmann

**Affiliations:** Conservative and Rehabilitative Orthopedics, Technische Universität München, Faculty for Sport and Health Science, Georg-Brauchle-Ring 60/62, D-80992 Munich, Germany; Institute of Health and Biomedical Innovation, Queensland University of Technology, Brisbane, Australia; Medical Park Bad Wiessee St. Hubertus, Bad Wiessee, Germany

## Abstract

**Background:**

Sensorimotor function is degraded in patients after lower limb arthroplasty. Sensorimotor training is thought to improve sensorimotor skills, however, the optimal training stimulus with regard to volume, frequency, duration, and intensity is still unknown. The aim of this study, therefore, was to firstly quantify the progression of sensorimotor function after total hip (THA) or knee (TKA) arthroplasty and, as second step, to evaluate effects of different sensorimotor training volumes.

**Methods:**

58 in-patients during their rehabilitation after THA or TKA participated in this prospective cohort study. Sensorimotor function was assessed using a test battery including measures of stabilization capacity, static balance, proprioception, and gait, along with a self-reported pain and function. All participants were randomly assigned to one of three intervention groups performing sensorimotor training two, four, or six times per week. Outcome measures were taken at three instances, at baseline (pre), after 1.5 weeks (mid) and at the conclusion of the 3 week program (post).

**Results:**

All measurements showed significant improvements over time, with the exception of proprioception and static balance during quiet bipedal stance which showed no significant main effects for time or intervention. There was no significant effect of sensorimotor training volume on any of the outcome measures.

**Conclusion:**

We were able to quantify improvements in measures of dynamic, but not static, sensorimotor function during the initial three weeks of rehabilitation following TKA/THA. Although sensorimotor improvements were independent of the training volume applied in the current study, long-term effects of sensorimotor training volume need to be investigated to optimize training stimulus recommendations.

**Trial registration:**

Clinical trial registration number: DRKS00007894

## Background

In the progression of osteoarthritis (OA), sensorimotor skills including proprioception [1, 2], static and dynamic balance [3], and neuromuscular control are known to degrade in response to pain avoidance and advancing inactivity. These sensorimotor deficiencies typically manifest as modified movement patterns and muscle weakness [4, 5] and have been shown to persist even after joint replacement. For instance, Thewlis et al. [6] observed persistent asymmetric load distribution in TKA patients 6 months after surgery and Levinger et al. [2] described proprioceptive deficits that remained for at least 12 months following TKA surgery. Similarly, Judd et al. [7] observed sensorimotor deficits following THA, with both strength and functional performance deficits persisting for at least one year after joint replacement.

Despite evidence that a full recovery of sensorimotor function is unlikely to occur within twelve months of THA or TKA [8], there is emerging evidence that sensorimotor function can be improved through dedicated sensorimotor training. For instance, Zech et al. [9] found that sensorimotor training improved dynamic balance in ankle sprain patients and resulted in a faster activation of hamstring muscles after a sudden perturbation of stance in patients with anterior cruciate ligament rupture. Similarly, sensorimotor training has been shown to produce positive effects on the response of hip OA and THA patients to sudden displacements [10], improve walking time and reduce knee reposition error in knee OA patients compared to strength training [11].

Along with muscular strengthening, joint flexibility training, and pain management, sensorimotor training has now become an integral part of rehabilitation guidelines following THA and TKA. However, evidence-based recommendations for sensorimotor training, particularly in post-operative rehabilitation programs, are currently lacking. Current guidelines are based mainly on anecdotal evidence and practical experience. Empirical evidence regarding the optimal sensorimotor training dose and the effects of training volume, frequency, duration, and intensity are still to be explored [1, 12–14].

The first purpose of the current study, therefore, was to quantify the progression of sensorimotor function during inpatient rehabilitation after THA and TKA. The second purpose was to evaluate the effects of sensorimotor training volume on sensorimotor function. We hypothesized that higher sensorimotor training volumes would improve sensorimotor function to a larger extent than lower training volumes.

## Methods

### Participants

Sixty-three consecutive patients presenting to an inpatient orthopaedic rehabilitation clinic (Medical Park St. Hubertus, Bad Wiessee, Germany) following TKA or THA to address unilateral joint disease were approached to participate in the study. Three patients declined to participate and two failed to meet the study inclusion criteria, which required patients to possess a minimum passive knee mobility between 30° and 85° knee flexion (85°/30°/0°, neutral zero method [15]) and to be able to fully weight-bear without aid for at least 30 s. Consequently, fifty-eight (29 males, 29 females) patients with unilateral TKA (*n* = 21) or THA (*n* = 37) participated in this study (Table 1). All patients were otherwise healthy and free of gross orthopaedic conditions of the lower limbs. Patients were randomly assigned to one of three groups, which differed only in the volume of sensorimotor training: two sessions per week (*n* = 20), four sessions per week (*n* = 15) and six sessions per week (*n* = 23). Base-line (pre-training) measurements took place 13.5 ± 2.8 days, on average, after surgery. All patients provided written informed consent, following a verbal and written explanation of the study procedures, which were approved by the ethics committee of the Technische Universität München, Germany.

**Table 1:** Demographic data (mean ± 1SD) of the treatment groups

Training volume	Two sessions	Four sessions	Six sessions
n	20	15	23
Age (years)	63.3 ± 10.3	61.1 ± 9.7	57.5 ± 15.2
Height (cm)	171.6 ± 10.7	174.5 ± 10.3	172.5 ± 7.5
Weight (kg)	79.2 ± 16.2	82.5 ± 18.8	86.4 ± 16.8
Days post op (days)	14.0 ± 2.4	13.3 ± 2.1	13.2 ± 3.5
Male/Female (%)	50 / 50	60 / 40	44 / 56
TKA/THA (%)	40 / 60	27 / 73	39 / 61

Between-group analysis (ANOVA) showed no significant differences (*p* > .05)

*TKA* total knee arthroplasty, *THA* total hip arthroplasty

### Intervention

All patients underwent three weeks of a standard rehabilitation protocol, which included exercise training, physical therapy, seminars, and educational group therapy. Within the standard rehabilitation protocol, patients also received a sensorimotor training program that included supervised exercise sessions involving three different therapeutic devices: (1) a balance pad (Balance Pad, Airex, Germany), (2) a ball cushion (Aero-Step® XL, Togu, Germany), and (3) a Proprio-Swing-System (systemreha GmbH & CO. KG, Germany). On each device, all sensorimotor exercises were conducted during quiet bipedal stance but the level of difficulty progressed from an ‘eyes open’ condition in the first week, through a ‘forward and backward leaning’ condition (within self-perceived limits of balance) during the second week and concluded with an ‘eyes closed’ condition in the third week. Sensorimotor exercises were undertaken for thirty seconds on each device, and were repeated six times within each training session. A thirty second rest period was provided between repetitions. Thus, in total, each sensorimotor training session lasted approximately 18 min including rest periods. In the regular rehabilitation protocol, the sensorimotor training session was scheduled six times per week. For this study three groups were established by adjusting the training volume from six, to four, and two sensorimotor training sessions per week.

### Procedure

Self-reported pain and function along with measures of stabilization capacity, static balance, proprioception and gait analysis were used as primary outcome measures. Outcome measures were taken at baseline (pre), and repeated after 1.5 weeks (mid) and at the conclusion of the 3 week program (post).

#### Gait analysis

Preferred over-ground walking speed was determined over a distance of 13 m [16] using two double light barriers (TDS lightbarriers, Werthner Sport Consulting KG, Austria). Step length was measured over the central 5 m of the walkway using an OptoGait System (OptoGait, Microgate, Italy) with a spatial resolution of 1.04 cm and a sampling frequency of 1000 Hz. In the event, that a patient was unable to walk without walkers, step length was not measured.

#### Stabilization capacity

Stabilization capacity was measured during bipedal stance on an oscillatory platform (Posturomed, Haider Bioswing, Germany) [10] that incorporated a provocation unit and a MicroSwing measuring system (three-dimensional acceleration sensor, Haider Bioswing, Germany). The provocation unit allowed for the precise displacement, fixation and the controlled release of the oscillatory platform. Patients were thereby exposed to a standardized horizontal unidirectional oscillatory stimulus and instructed to dampen the movement of the platform as quickly as possible to return to quite standing. Acceleration of the platform was measured over ten seconds and the procedure was repeated three times, with oscillations independently induced in both the medio-lateral and anterior-posterior directions. Proprietary software was subsequently used to calculate the stability index for each trial. The dimensionless index, which reflects the patient’s capacity to stabilize the oscillatory platform, ranged from 0 to 1000 with higher scores representing higher stabilization capacity. Average stability indices were calculated from the three trials undertaken in each direction to give rise to each patients’ anterior-posterior and medio-lateral stabilization capacity.

#### Static balance

Static balance was assessed using previously published methods [17]. In brief, displacement of the centre of pressure was recorded while patients stood as still as possible on a pressure platform (footscan® USB plate, RSscan International, Belgium) under four sequential experimental conditions; (1) bipedal stance with eyes open, (2) bipedal stance with eyes closed, (3) semi-tandem stance with the operated leg positioned anteriorly, and (4) semi-tandem stance with the operated leg positioned posteriorly. Balance data for each experimental condition were collected for 20 s at a sampling rate of 43.3 Hz [3]. For each trial, the root mean square (RMS) of the displacement of the centre of pressure (COP) was calculated in both the medio-lateral and anterior-posterior directions and used in subsequent analysis.

#### Proprioception

Knee joint proprioception was assessed using the passive-active angle-reproduction test [18], conducted at target angles of 40° and 60° of knee flexion. Patients were seated on a height adjustable therapy chair with the knee of the operated leg positioned at 90 degrees of flexion. The foot was positioned on a low friction linear bearing, so that active and passive movement of the knee could be accomplished with minimal effort. A digital goniometer (accuracy: 0.1°, digital angle rule 200 mm, Trend, United Kingdom) was attached to the lateral aspect of the knee using Velcro straps with the angular point device positioned over the estimated joint centre. Patients were instructed to close their eyes throughout proprioception measurement. From the initial position of 90 degrees of flexion, the knee was then passively moved to a target angle of either 40 or 60 degrees. The target angle was maintained for four seconds before the knee was passively returned to the initial position. Patients were then requested to actively move their leg to reproduce the target angle. The absolute difference between the actively reproduced angle and the target angle was subsequently calculated and used for further analysis.

#### Functional assessment

The German adaptation of the Lequesne Algofunctional Questionnaire [19] was used to assess self-perceived functional impairment, stiffness, and pain during activities of daily living. The questionnaire consisted of 11 items analysing pain (5 items), maximum walking distance (2 items) and activities of daily living (4 items). Scores can range from 0 to 24 and were subclassified according to the criteria of Nilsdoter, where a score of 0 represents “no handicap”, 1 – 4 reflects “mild handicap”, 5–7 represents “moderate handicap”, 8 – 10 reflects “severe handicap”, 11 – 13 represents “very severe handicap”, and a score ≥ 14 indicates an “extremely severe handicap” [20]. The questionnaire takes approximately two minutes, on average, to complete and has been shown to have good acceptance among patients [19]. The use of pain-modifying medication was recorded as a dichotomous variable prior to each measurement.

#### Statistical analysis

The Statistical Package for the Social Sciences (version 21, IBM, USA) was used for all statistical procedures. Kolmogorov–Smirnov tests were used to evaluate data for underlying assumptions of normality. Outcome variables were determined to be normally distributed, and consequently means and standard deviations have been used as summary statistics. Between–group differences in age and body anthropometry were investigated using one-way analysis of variance (ANOVA). The effect of time (pre, mid, post) and training volume (2, 4 or 6 sessions per week) on measures of static balance, proprioception and basic gait parameters were evaluated using two–way repeated measures ANOVA in which time (pre, mid, post) was treated as a within–subject factor. Significant effects for time were evaluated using *post hoc* paired t-tests. Partial effect size (*η*_*p*_^2^) was calculated as an estimate of effect size. An alpha level of .05 was used for all univariate tests of significance.

## Results

One-way ANOVA demonstrated no difference between the three groups with respect to age, height and body weight at baseline (Table 1).

### Gait analysis

**Fig. 1:**
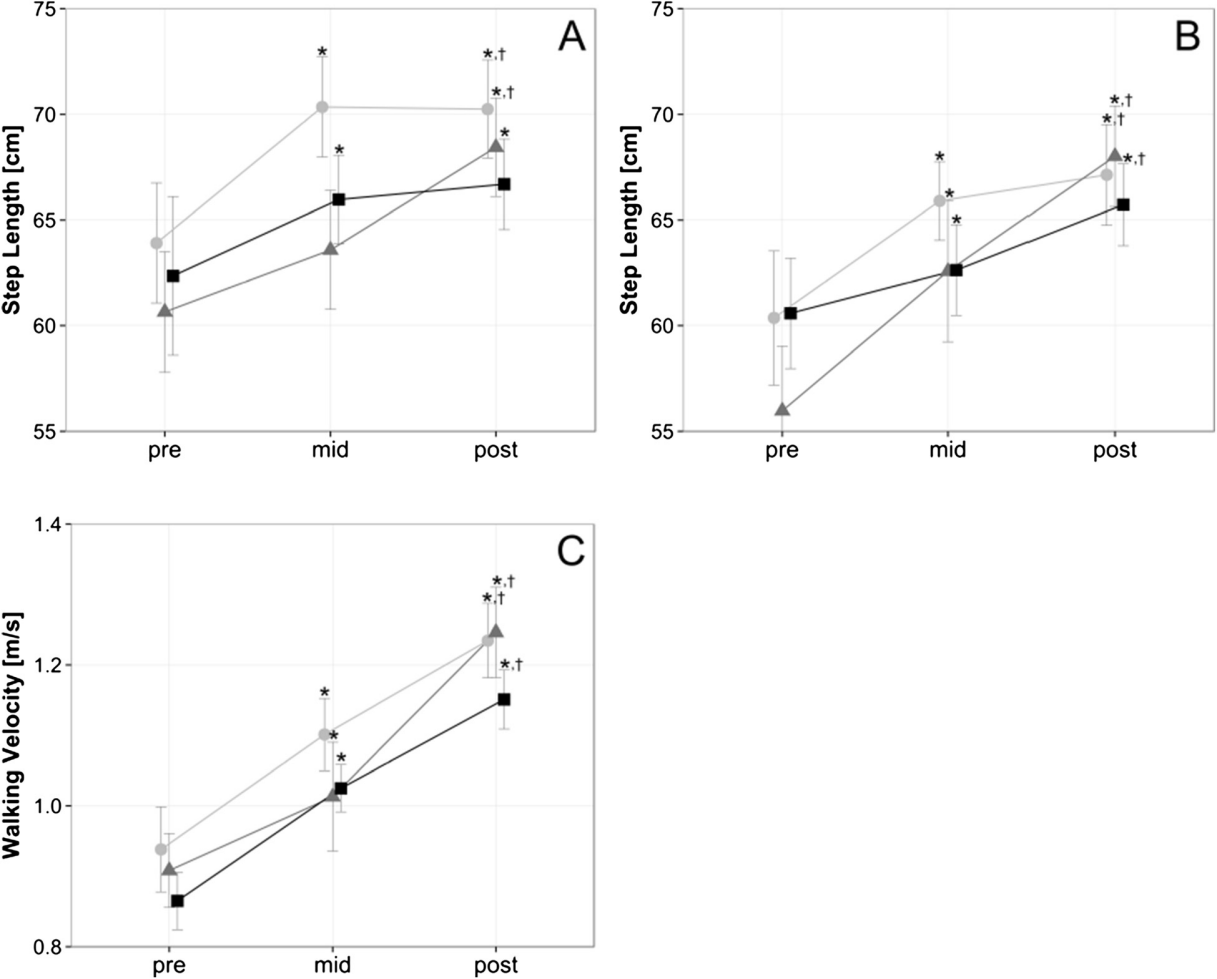
Gait Analysis. Changes in gait analysis parameter (**a**) step length operated leg, (**b**) step length not operated leg, (**c**) walking velocity). Data are mean ± 1 SE for the three experimental groups (light circle = 2 weekly sessions, grey triangle = 4 weekly sessions, black sqaure = 6 weekly sessions). * *P* ≤ 0.05 vs. pre, † *P* ≤ 0.05 vs. mid

Walking velocity significantly increased over time (*p* < .001; *η*_*p*_^2^=.670), but did not differ between training volumes (*p* = .481) (Fig. 1). Similarly, step length increased in the operated (*p* < .001, *η*_*p*_^2^ = 0.549) and non-operated leg (*p* < .001, *η*_*p*_^2^ = 0.630) over time, but was not significantly different between training volumes (operated leg, *p* = .497; not operated leg, *p* = .559).

### Stabilization capacity

**Fig. 2:**
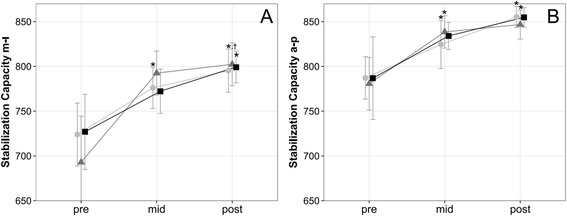
Stabilization Capacity. Changes in capability to restabilze a sudden pertubation of the underground (**a**) stabilization capacity medio-lateral (m-l), (**b**) stabilization capacity anterior-posterior (a-p)). Data are mean ± 1 SE for the three experimental groups (light circle = 2 weekly sessions, grey triangle = 4 weekly sessions, black sqaure = 6 weekly sessions). * *P* ≤ 0.05 vs. pre, † *P* ≤ 0.05 vs. mid

Although the stability index significantly increased over time in both the anterior-posterior (*p* < .001, *η*_*p*_^2^ = 0.184) and medio-lateral (*p* < .001, *η*_*p*_^2^ = 0.203) directions (Fig. 2), there was no significant difference between training volumes (anterior-posterior *p* = .942; medio-lateral *p* = .845).

### Static balance

**Fig. 3:**
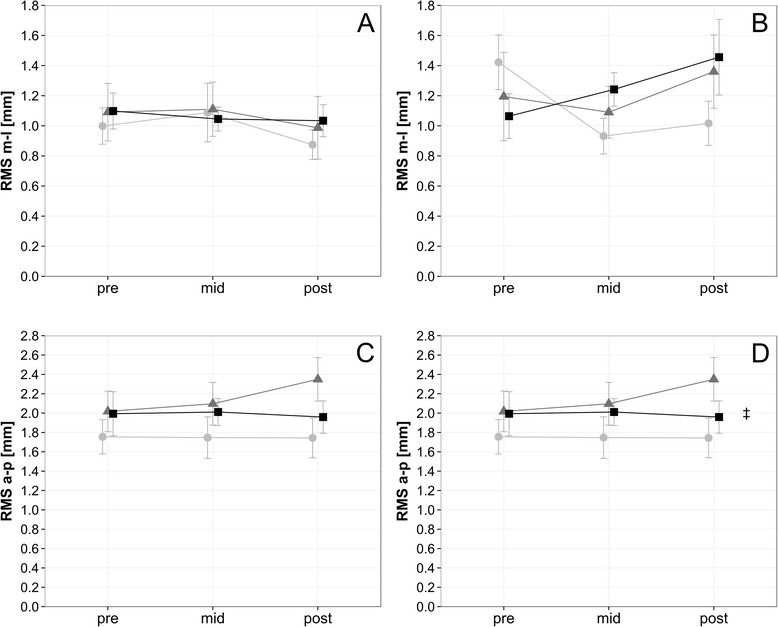
Bipedal Stance. Course of static balance measurement with an unsystematic interaction between time and training volume in the bipedal stance with eyes closed condition (**a**) RMS medio-lateral bipedal stance with eyes open, (**b**) RMS medio-lateral bipedal stance with eyes closed, (**c**) RMS anterior-posterior bipedal stance with eyes open, (**d**) RMS anterior-posterior bipedal stance with eyes closed). Data are mean ± 1 SE for the three experimental groups (light circle = 2 weekly sessions, grey triangle = 4 weekly sessions, black sqaure = 6 weekly sessions). ‡ *P* ≤ 0.05 ANOVA interaction

**Fig. 4:**
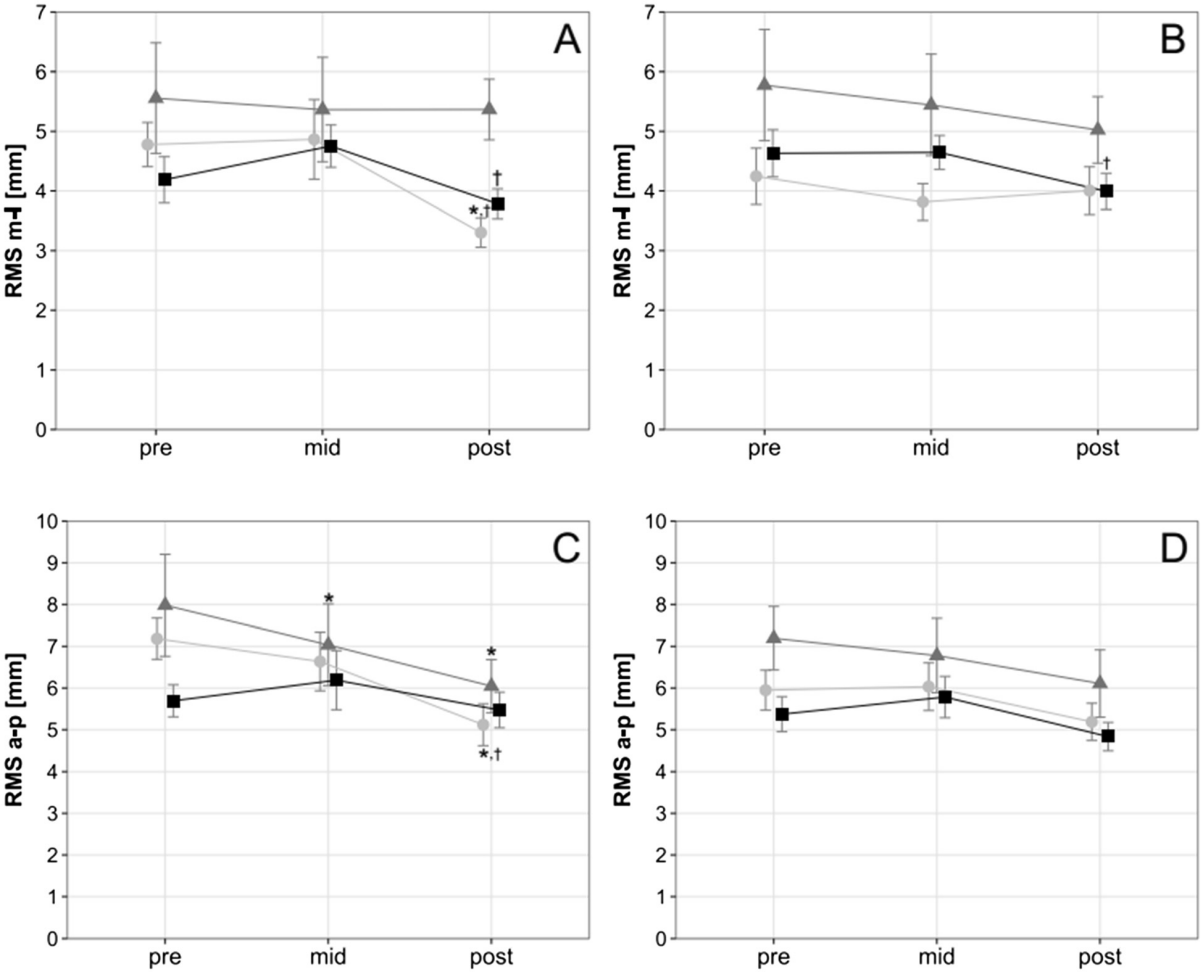
Semitandem Stance. Course of static balance measurement in semitandem stance conditions (**a**) RMS medio-lateral semitandem stance with operated leg positioned in front, (**b**) RMS medio-lateral semitandem stance with not-operated leg positioned in front, (**c**) RMS anterior-posterior semitandem stance with operated leg positioned in front, (**d**) RMS anterior-posterior semitandem stance with not-operated leg positioned in front). Data are mean ± 1 SE for the three experimental groups (light circle = 2 weekly sessions, grey triangle = 4 weekly sessions, black sqaure = 6 weekly sessions). * *P* ≤ 0.05 vs. pre, † *P* ≤ 0.05 vs. mid

There were no significant main effects of time or training volume on two of the four static balance conditions. There was a non-systematic though significant interaction between time and training volume in the RMS of the anterior-posterior displacement of the COP during the eyes closed condition (*p* = .033; *η*_*p*_^2^ = 0.093), Fig. 3). In semi-tandem stance conditions, the RMS decreased significantly over time in both the anterior-posterior and medio-lateral directions when the operated leg was positioned anteriorly (anterior-posterior: *p* = .003, *η*_*p*_^2^ = 0.119); medio-lateral: *p* = .03, *η*_*p*_^2^ = 0.074) but decreased only in the anterior-posterior direction when the operated leg was positioned behind the non-operated leg (*p* = .009, *η*_*p*_^2^ = 0.011, Fig. 4).

### Proprioception

**Fig. 5:**
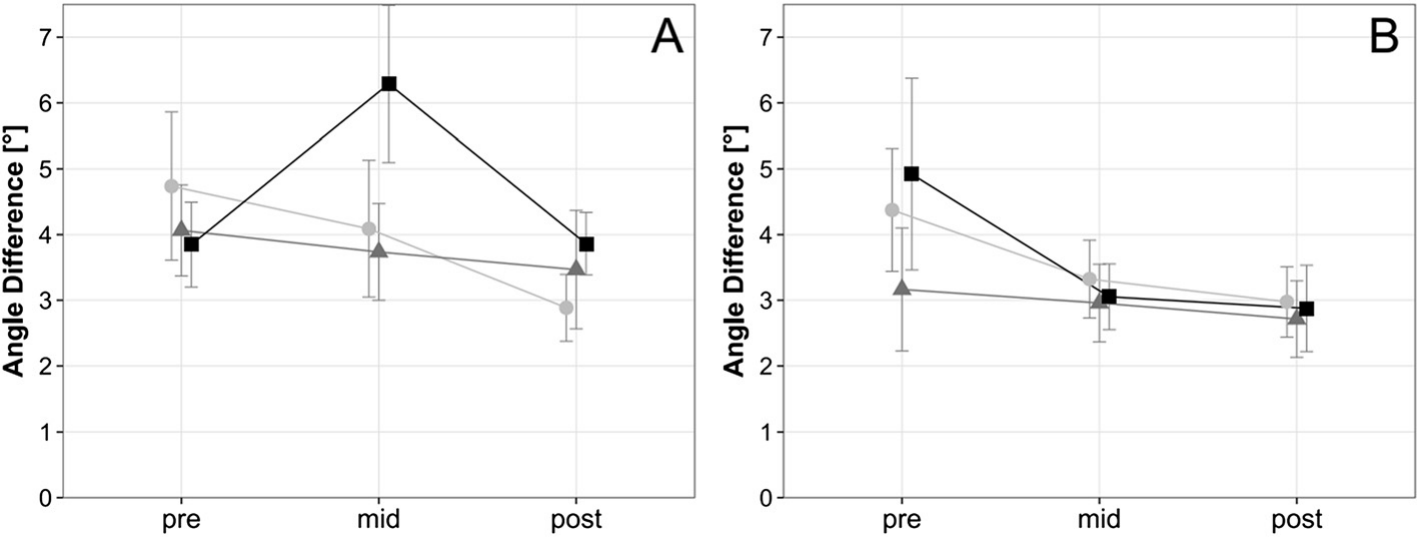
Proprioception. Proprioception measured by angle reproduction test (**a**) target angle 40° knee flexion, (**b**) target angle 60° knee flexion). Data are mean ± 1 SE for the three experimental groups (light circle = 2 weekly sessions, grey triangle = 4 weekly sessions, black sqaure = 6 weekly sessions)

There was no significant difference in the angle reproduction test at either target angle over time or between training volumes (Fig. 5).

### Functional assessment

**Fig. 6:**
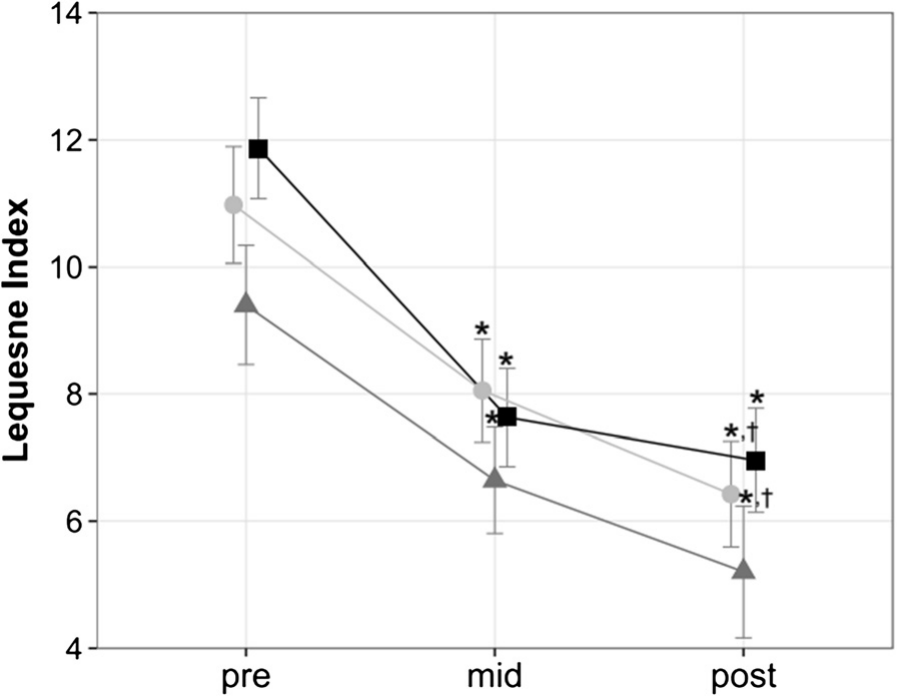
Self-Assessment. Changes in Lequesne Algofunctional Index in course of in-patient rehabilitation. Data are mean ± 1 SE for the three experimental groups (light circle = 2 weekly sessions, grey triangle = 4 weekly sessions, black sqaure = 6 weekly sessions). * *P* ≤ 0.05 vs. pre, † *P* ≤ 0.05 vs. mid

Self-reported function scores improved significantly over time (*p* < 001, *η*_*p*_^2^ = 0.584) but did not differ between training volumes (*p* = .458) (Fig. 6).

## Discussion

The first purpose of the study was to quantify the progression of sensorimotor function during inpatient rehabilitation using a test battery that included static and dynamic measures of sensorimotor function. We could observe improvements in gait parameters, postural stability and in self-reported function during the three week period of early recovery in THA and TKA patients. The improvements in walking velocity (for all groups ∆_post-pre_ > +0.25 m/s) are considered to reflect a clinically meaningful change [21].

We observed significant improvements in stabilization capacity over the three week rehabilitation period. As sensorimotor training is known to improve the reaction of individuals to sudden disturbances of the support surface [10], we attribute a major contribution to the improved stabilization capacity of our patients to sensorimotor training but recognise potential time or learning effects may also play a role. While our results are consistent with those reported by Boeer et al. [22], we evaluated stabilization capacity during bipedal, rather than unipedal, stance since the majority of participants in our study were unable to stand on one leg without aid.

In contrast to the improvements in stabilization capacity, static balance improved only in the more challenging semi-tandem stance conditions (operated leg in front or behind). While the present experimental setup did not allow for a mechanistic explanation as to why control of quiet bipedal stance did not improve during rehabilitation periods, asymmetric load distribution is known to increase COP displacement during quiet stance and has been shown persist in TKA patients for at least six months following surgery [6, 23]. In light of the magnitude of load asymmetry that occurs following THA [24], however, this effect is likely too low to explain the impairment in postural control observed in the current study [25]. Thus, our findings suggest that recovery of normal bipedal stance control is not improved with sensorimotor training and likely needs substantial time for recovery to occur, if at all. Semi-tandem stance conditions cause between 258–319 % (anterior-posterior) and 350–355 % (medio-lateral) more postural sway as compared to bipedal stance with open eyes at baseline. It remains questionable, whether improvements in these more challenging balance conditions are achieved through improved intra- and inter-muscular coordination or better sensorimotor control in general.

Proprioception, as defined by the angle reproduction measurement, showed no significant changes in any group over time. A trend towards an improvement can be seen at a target angle of 60°, however this was not statistically significant. For most of the TKA patients, particularly at baseline, replication of the 40° target angle was close to the upper limit of the available range of motion of the knee and was often coupled with pain. Thus, pain may have confounded measurements of proprioception in the current study and may also, in part, account for the inconsistent findings reported elsewhere in the recovery of joint-position sense in THA and TKA patients following surgery [26, 27]. While improvements have been reported by some studies following TKA [26], others have observed persistent deficits for up to twelve months following TKA [8].

The second purpose of the study was to evaluate the effects of sensorimotor training volume on sensorimotor function. In contrast to our hypothesis, we found that decreasing the training volume of sensorimotor training to fewer than six sessions per week had no significant effect on sensorimotor function in our cohort. There are several possible explanations for this observation.

First, it is possible that the sensorimotor training program may not affect the recovery of sensorimotor function during in-patient rehabilitation. However, other studies have shown that sensorimotor function improves with sensorimotor training during recovery from ankle sprain [9], following anterior cruciate ligament rupture [9], with knee osteoarthritis [27], TKA [13], and following THA [10].

Second, the training volume employed in the current study may not have been sufficient to induce neuromuscular adaptation. In the absence of recommendations on the intensity of sensorimotor training, however, the duration of the training program employed in the current study was designed to fall within the range that has been previously shown to have beneficial effects [28, 29].

Finally, while there is some evidence that increasing training to more than one session per week invokes additional sensorimotor benefit [29], it is possible that there is a ceiling effect, in which there is no additional benefit beyond two sensorimotor training sessions per week. It remains to be shown whether, in the course of further rehabilitation of THA or TKA, a higher training frequency leads to greater improvement in sensorimotor function.

This study has several limitations which should be considered when interpreting the results. First, pain sensation is known to influence proprioception [30], and by the patients’ general pain sensitivity, surgical outcome, and level of pain medication. During the course of our study, pain medication was reduced progressively on an individual basis, and hence might have influenced the sensorimotor function at different time points. Evidence of an effect of pain on sensorimotor function, however, is contradictory [30] and we observed no differences in the use of pain medication between groups. Moreover, despite a reduction in self-reported pain in our cohort over time, we observed no significant change in proprioception performance. Second, repeated measurements carry the risk of potential learning effects. To keep potential learning effects to a minimum, patients were exposed to the measurement devices for as short as possible and were not permitted to use the devices between measurements. Finally, there may be a temporal delay in the effects of training on sensorimotor performance. Previous research, however, has shown improvements in dynamic balance tasks and structural reorganization of grey and white matter after as little as two 45-min training session within two weeks [31]. Despite these limitations, we believe this study provides clinically relevant insights into the progress of sensorimotor function and the effects of sensorimotor training volume during the early recovery following total hip or knee arthroplasty. Further research investigating potential differential effects of sensorimotor training on TKA and THA patients over a longer duration of recovery is warranted.

## Conclusion

We were able to quantify improvements in measures of dynamic, but not static, sensorimotor function during the initial three weeks of recovery from TKA or THA. Sensorimotor improvements were independent of sensorimotor training volume, as sensorimotor performance did not differ with weekly training volumes of two, four or six sessions. Thus, in contrast to common clinical practise, greater volume of sensorimotor training during rehabilitation does not necessarily lead to better sensorimotor function. Further research investigating the effect of training volume and its long-term effects are needed, however, before definitive recommendations regarding optimal training stimulus (magnitude, frequency, duration) can be formulated.
